# Biomarkers of Periodontal Tissue Remodeling during Orthodontic Tooth Movement in *Mice and Men*: Overview and Clinical Relevance

**DOI:** 10.1155/2013/105873

**Published:** 2013-04-23

**Authors:** Fabrizia d'Apuzzo, Salvatore Cappabianca, Domenico Ciavarella, Angela Monsurrò, Armando Silvestrini-Biavati, Letizia Perillo

**Affiliations:** ^1^Department of Orthodontics, Second University of Naples, Via L. De Crecchio 6, 80138 Naples, Italy; ^2^Department of Radiology, Second University of Naples, Piazza L. Miraglia 5, 80138 Naples, Italy; ^3^Department of Clinical and Experimental Medicine, University of Foggia, Viale L. Pinto, 71100 Foggia, Italy; ^4^Department of Orthodontics, University of Genoa, Corso Europa 35, 16132 Genoa, Italy

## Abstract

Biologically active substances are expressed by cells within the periodontium in response to mechanical stimuli from orthodontic appliances. Several possible biomarkers representing biological modifications during specific phenomena as simile-inflammatory process, bone resorption and formation, periodontal ligament changes, and vascular and neural responses are proposed. Citations to potentially published trials were conducted by searching PubMed, Cochrane databases, and scientific textbooks. Additionally, hand searching and contact with experts in the area were undertaken to identify potentially relevant published and unpublished studies. Selection criteria were as follows: animal models involving only mice and rats undergoing orthodontic treatment; collection of gingival crevicular fluid (GCF) as a noninvasively procedure for humans; no other simultaneous treatment that could affect experimental orthodontic movement. The data suggest that knowledge of the remodeling process occurring in periodontal tissues during orthodontic and orthopedic therapies may be a clinical usefulness procedure leading to proper choice of mechanical stress to improve and to shorten the period of treatment, avoiding adverse consequences. The relevance for clinicians of evaluating the rate of some substances as valid biomarkers of periodontal effects during orthodontic movement, by means of two models of study, *mice and men*, is underlined.

## 1. Introduction

**Figure 1:**
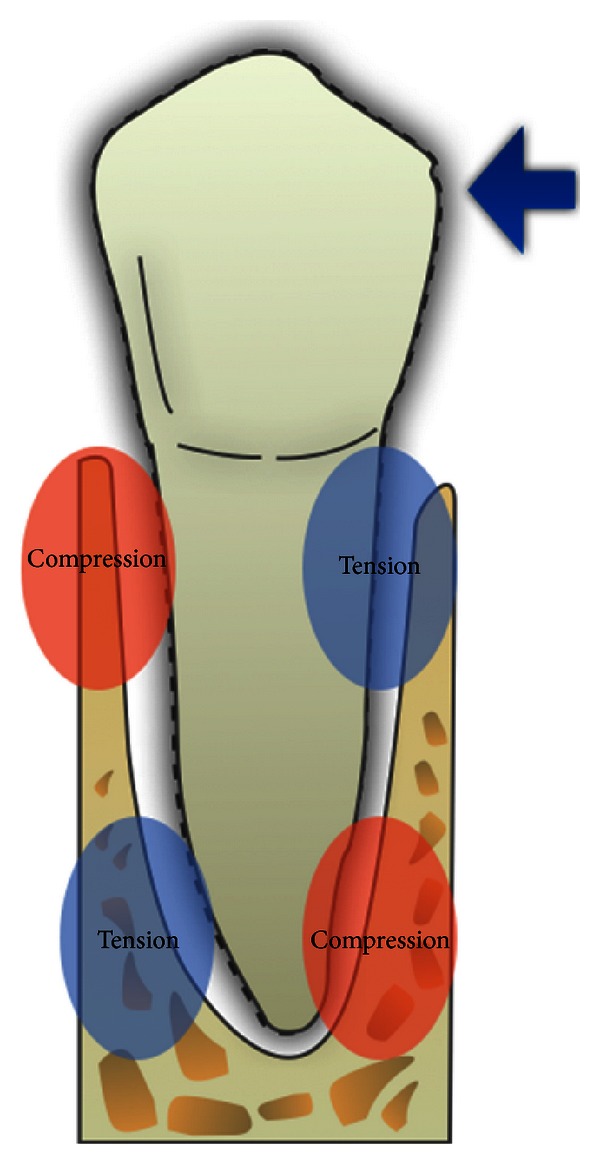
Tension and pressure sites by application of an orthodontic force.

**Figure 2:**
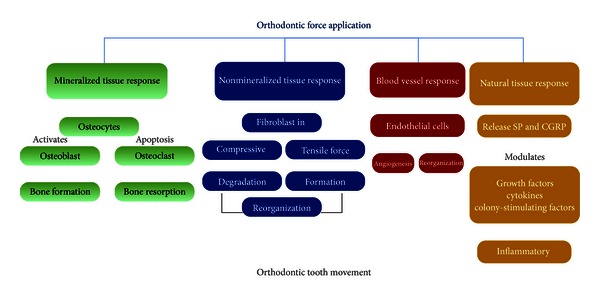
Effects of orthodontic force application on mineralized and nonmineralized paradental tissues.

Tooth movement by orthodontic force application is dependent on remodeling in periodontal ligament and alveolar bone. Orthodontic movement should be described as a continual and balanced process characterized by bone deposition and bone resorption, respectively, on pressure and tension sites (Figure 1). Orthodontic forces by virtue of altering the blood flow and localized electrochemical environment upset the periodontal space. These abrupt alterations lead to the generation and propagation of signaling cascades and associated tissue remodeling by delineating biochemical and cellular reactions occurring in mineralized (alveolar bone) and nonmineralized (periodontium) paradental tissues (Figure 2). Several possible biomarkers representing these biological modifications are expressed during specific phenomena, that is, simile-inflammatory process, bone resorption and formation, periodontal ligament changes, and vascular and neural responses [1].

To identify the degree of remodeling occurring in the periodontal tissues during orthodontic or orthopedic retention by monitoring the levels of certain biochemical mediators may be a clinically useful procedure, because of their important roles in tooth movement and even in tissue damage.

In this paper, the importance of evaluating the levels of substances as valid biomarkers of periodontal effects of an orthodontic treatment is emphasized, through an accurate description of the specific role of each of them.

Citations to potentially published trials were conducted by searching PubMed, Cochrane databases, and scientific textbooks. Additionally, hand searching and contact with experts in the area were undertaken to identify potentially relevant published and unpublished studies. Selection criteria were as follows: animal models involving only mice and rats undergoing orthodontic treatment; collection of gingival crevicular fluid (GCF) as a noninvasively procedure for humans; no other simultaneous treatment that could affect experimental orthodontic movement.

**Figure 3:**
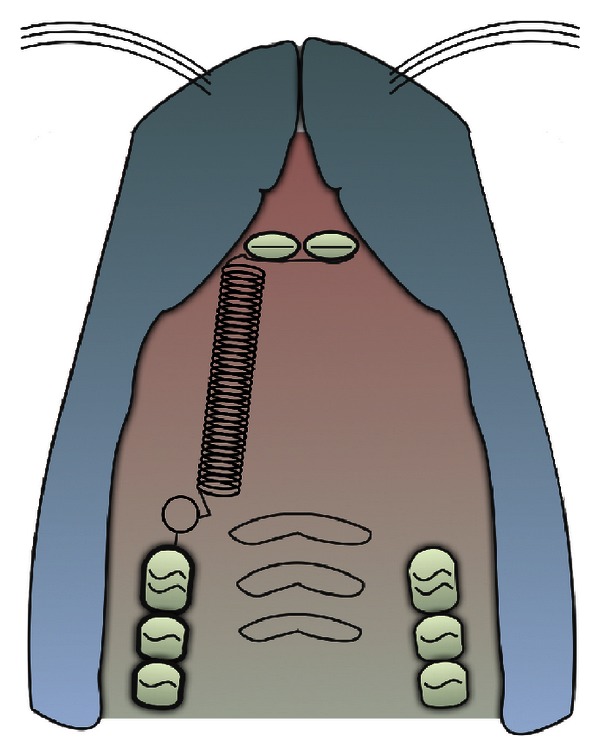
Mice and rats model of experimental orthodontic tooth movement.

**Figure 4:**
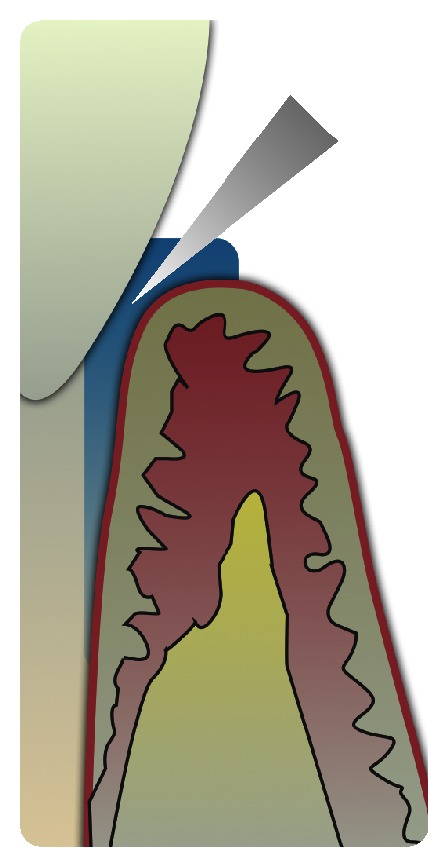
Human gingival crevicular fluid.

Only studies on mice and rats have been considered as animal models (Figure 3), whereas changes in the composition of gingival crevicular fluid (GCF) (Figure 4) during orthodontic and orthopedic tooth movement have been selected in order to monitor the expression of these biomarkers noninvasively in humans. In fact, substances involved in bone remodeling are produced by periodontal ligament cells in sufficient quantities to diffuse into GCF.

Thus, by means of the two models of study, *mice and men*, the clinical usefulness of some biomarkers in orthodontics is properly analyzed.

## 2. Biomarkers of Periodontal and Bone Responses to Orthodontic Force Application

Biological mechanisms that control the shift from the stimulus, consisting of a continuous force application, to the reaction, represented by tooth displacement in periodontal space, can be evaluated by considering the theory of the pressure tension, according to which cell differentiation and subsequent tooth movement are controlled by chemical signals. The sequence of events following orthodontic tooth movement can be characterized using suitable biomarkers.

### 2.1. Proinflammatory Cytokines: Interleukin-1*β* (IL-1*β*), Interleukin-6 (IL-6), Interleukin-8 (IL-8), Tumor Necrosis Factor-*α* (TNF-*α*), and Prostaglandin E (PGE)

In experimental tooth movement, the presence of neuroimmune interactions may be of primary importance in the initial inflammatory response [2]. Interleukin-1*β* (IL-1*β*) is one of the most potent cytokines in periodontal environment during the initial stage of orthodontic tooth movement [3]. The potential sources of IL-1*β* during tooth movement include cells such as fibroblasts, macrophages, cementoblasts, cementoclasts, osteoblasts, and osteoclasts [4]. In the early stages of tooth movement (at 12 and 24 hours), many periodontal cell types stained positively for IL-1*β* [5]. Particularly, IL-1*β* is secreted by osteoclasts as an immediate response to mechanical stress during the initial stage of orthodontic treatment, and at later stages by macrophages, whose accumulation has been observed in compressed areas. Since survival, fusion, and activation of osteoclasts correlate with IL-1*β*, this Interleukin also determines the amount of tooth movement dependently on the efficiency of alveolar bone remodeling process [6]. The concentration of IL-1*β* mRNA in rats' periodontal ligament after orthodontic force loading is increased within 3 h, particularly on pressure side [7]. An inducted inflammatory process by perforating the buccal cortical plate of orthodontically treated rats showed higher levels of expression of inflammatory cytokines and their respective receptors [8]. In fact, administration of exogenous IL-1 Receptor Antagonist (IL-1RA) to treated mice leads to a 66% decrease in the levels of IL-1*β* when compared to the experimental tooth movement of vehicle treated mice, indicating a reduction of the number of osteoclasts on pressure side of periodontal tissues after histological characterization and a consequent downregulation of orthodontic tooth displacement rate in mice treated with IL-RA therapy [9]. Moreover, there is a relationship between velocity of tooth translation and IL-1*β* gene cluster polymorphisms evaluated in GCF [10].

**Figure 5:**
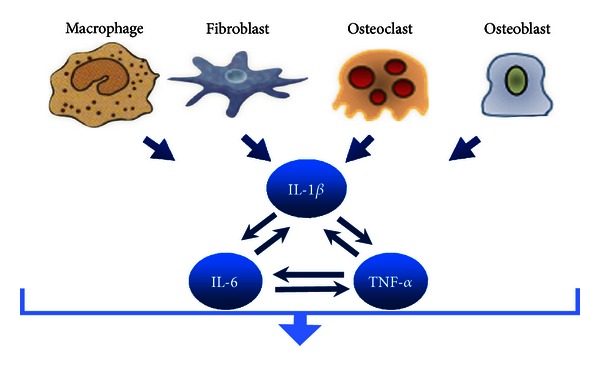
IL-1*β* cellular sources and its interactions with IL-6 and TNF-*α* in osteoclastic cell activation.

IL-1*β* is also considered to be a powerful inducer of Interleukin-6 (IL-6) production; it overlaps with IL-6 and Tumor Necrosis Factor-*α* (TNF-*α*) in their actions (Figure 5) [11]. IL-6 has an increased amount after 24 hrs [12]. It regulates immune responses in inflammation sites [13], and it has an autocrine/paracrine activity stimulating osteoclast formation and bone-resorbing activity of preformed osteoclasts [14].

TNF-*α* is another proinflammatory cytokine shown to elicit acute or chronic inflammation and stimulate bone resorption, by means of the direct differentiation of osteoclast progenitors to osteoclasts in the presence of macrophage-colony-stimulating factor [15].

Tuncer et al. [16] reported increased levels of Interleukin-8 (IL-8) at PDL tension sites and proposed it to be a triggering factor for bone remodeling. In human gingival crevicular fluid many findings have confirmed increased levels of these proinflammatory cytokines involving in parodontal remodeling during orthodontic tooth movement [12, 17, 18].

Clinical and animal studies by various authors have identified prostaglandins E (PGE_1_ and PGE_2_) role in stimulating bone resorption [19, 20]. A primary response to the pressure stimulus is the liberation of prostaglandins; it occurs when cells are mechanically deformed and the consequent mobilization of membrane phospholipids leads to the formation of inositol phosphate, an important chemical messenger. PGE_2_, specially, is able to mediate inflammatory responses and induces bone resorption by activating osteoclastic cells [21]. The literature reports that prostaglandins directly stimulate osteoclast production and their capacity to form ruffled border and effect bone resorption. In addition, the GCF level of PGE_2_ reflect the biologic activity in periodontium during orthodontic tooth movement, and it is significantly higher in both tension and compression sides [22].

### 2.2. RANK/RANKL/Osteoprotegerin (OPG) System

A variety of proliferation markers are expressed during orthodontic movement. The increase of differentiated osteoblasts in tension areas is indicated by Runx2 [1], 3.6Col1-GFP, and BSP-GFP expression cells [23], whereas KI-67 and RANKL (receptor activator of nuclear factor-kappaB ligand) [24, 25] indicate the recruitment of osteoclasts in compression areas. Of note, the TNF-related ligand, RANKL and its decoy receptor, RANK (receptor activator of nuclear factor-kappaB), and Osteoprotegerin (OPG) were found to play important roles in bone metabolism regulation. RANKL is a downstream regulator of osteoclast formation and activation, through which many hormones and cytokines produce their osteoresorptive effect [26]. RANKL is expressed on osteoblast cell lineage and exerts its effect by binding the RANK receptor on osteoclast cell lineage. This binding leads to rapid differentiation of hematopoietic osteoclast precursors to mature osteoclasts. OPG is a decoy receptor produced by osteoblastic cells, which competes with RANK for RANKL binding. Biologic effects of OPG on bone cells include inhibition of terminal stages of osteoclast differentiation, suppression of activation of matrix osteoclasts, and induction of apoptosis. Thus, bone remodeling is controlled by a balance between RANK-RANKL binding and OPG production. Kanzaki et al. [27] reported recently that OPG gene transfer produces in periodontal tissues an inhibited RANKL-mediated osteoclastogenesis and experimental tooth movement in rats. This finding demonstrates the potential value of employing a biological agent as an adjunct of orthodontic treatment. Thus, the inhibition of the activity of RANKL in its promoting osteoclast differentiation could be very helpful in preventing movement of anchor teeth during orthodontic treatment, and a relapse during the posttreatment period.

### 2.3. Macrophages-Colony-Stimulating Factors (M-CSF)

Colony-stimulating factors (CSF) are specific glycoproteins, which interact to regulate production, maturation, and function of monocyte-macrophages (M-CSF) as well as granulocytes (G-CSF). They might have implications in bone remodeling and thereby during tooth movement [21]. An important implication in tooth movement is played by the M-CSF through an increased early osteoclastic recruitment and differentiation [28]. In the future, optimal dosages of M-CSF, already correlated with measureable changes in tooth movement and gene expression, will provide a great potential in accelerating clinically the rate of tooth movement.

### 2.4. Vascular Endothelial Growth Factor (VEGF) as a Key Factor of Neovascularization

Vascular endothelial growth factor (VEGF) is a cytokine involved in tissue neoformation, since it increases vascular permeability and mediates angiogenesis [29]. During orthodontic tooth movement, compressive forces induce formation of new blood vessels in periodontium with the activation of VEGF [30]. The localization of VEGF was performed *in vivo *during experimental tooth movement in rat periodontal tissues with immunohistochemical analysis. VEGF immunoreactivity was found in vascular endothelial cells, in fibroblasts adjacent to hyalinized tissue, a local necrotic area in compressed zone, in osteoblasts and osteoclasts, and in mononuclear cells. Kaku et al. detected VEGF mRNA in fibroblasts and osteoblasts in tension area of mice periodontal ligament during experimental tooth orthodontic movement [31, 32]. These investigations demonstrate the VEGF role in remodeling periodontal ligament and in bone resorption and formation.

### 2.5. Neuropeptides during Neural Tissue Response to Orthodontic Tooth Movement

During orthodontic tooth movement the increased concentration of biologically active proteins contributes to neurogenic inflammation occurring in periodontium. Somatosensory neurons transmit signals from periodontal peripheral nerve fibers to the central nervous system. With application of physiologic orthodontic force, periodontal peripheral nerve fibers release calcitonin gene related peptide (CGRP) and substance P. In addition to acting as neurotransmitters, CGRP and substance P serve as vasodilators, increasing vascular flow and permeability (diapedesis) and stimulating plasma extravasation and leukocyte migration into tissues (transmigration).

CGRP induces bone formation through osteoblast proliferation and osteoclast inhibition. CGRP receptors are found on osteoblasts, monocytes, lymphocytes, and mast cells, and receptor activation results in amplified intercellular communication, promoting cytokine (inflammatory mediator molecules) synthesis and release.

Normal periodontal and alveolar bone innervations are essential to orthodontic tooth movement-associated periodontal remodeling. Healthy innervation promotes maximum blood flow during orthodontic tooth movement, whereas denervation reduces blood flow and bone formation [33]. Substance P (SP), another sensory neuropeptide released from peripheral endings of sensory nerves, can modify the secretion of proinflammatory cytokines from immunocompetent cells during periodontal tissue remodeling. Noteworthy, SP stimulates PGE_2_ production [34].

### 2.6. Enzymes Reflecting Biological Activity in Periodontium: Caspase-1, *β*-Glucuronidase (*β*G), Aspartate Aminotransferase (AST), and Lactate Dehydrogenase (LDH)

Generally, a high enzyme activity is suggestive of a greater cellular activity [35]. An apoptotic process occurs to eliminate the hyalinized periodontal tissue formed during the early stages of orthodontic movement. Among mediators of the apoptotic response activated by alterations in the intracellular ionic milieu, the most relevant is caspase-1. It has the role of processing and activating proIL-1*β* and other proinflammatory cytokines. Caspase-1 mRNA expression is increased in a rat model undergoing orthodontic treatment, and its rate changes with different temporal phases of orthodontic tooth displacement [36]. Some studies demonstrate that, with an excessive local orthodontic force application or with a pathologic hyperexpression of caspase-1 in a subject affected by diseases like rheumatoid arthritis, an irreversible root resorption and a deformation of periodontal tissues might appear. So, the administration of molecules with the function of inhibiting caspase-1 activity (VX-765 and Pralnacasan) may be a method to preserve the structure of periodontal ligament [37, 38]. A biomarker of primary granule release from polymorphonuclear leukocytes is the lysosomal enzyme *β*-glucuronidase (*β*G). Increased levels of this enzyme have been found in the GCF of adolescents treated with rapid maxillary expander. Moreover, *β*G, as other biochemical mediators like IL-1*β*, responds to direct and indirect application of mechanical force to teeth, with an increased level that is higher than following stronger forces [39].

Aspartate aminotransferase (AST) and lactate dehydrogenase (LDH) activities in GCF have been measured to confirm the biological activity which occurs in the periodontium during orthodontic treatment. They are soluble enzymes normally confined to the cytoplasm of cells then released to the extracellular environment after cell necrosis. The GCF AST activity is significantly elevated in both tension and compression sites at days 7 and 14. This rise is explained as a consequence of a controlled trauma which produces cell death as a consequence of mechanical force exerted on alveolar bone and periodontal ligament. A low increase of GCF AST activity reflects the application of orthodontic force on teeth, particularly in dental sites undergoing pressure, while an occlusal trauma leads to a higher amount of enzymatic level [40]. It positively relates with compression sites caused by an orthodontic tooth movement [41].

### 2.7. Enzymes Involved in Bone Cells Activities: Alkaline Phosphatase (ALP) and Acid Phosphatase (ACP)

The biological response incident to orthodontic tooth movement ultimately involves alterations in the surrounding bone architecture [42]. Bone metabolism is associated with alkaline phosphatase (ALP) and acid phosphatase (ACP), expressed, respectively, by osteoblasts and osteoclasts. ALP is an ubiquitous tetrameric enzyme, localized outside cell membrane [43]. ALP activity is found at much higher levels in periodontal ligament than in other connective tissues [44]. As a result of orthodontic force application, these enzymes, produced in the periodontium, diffuse into the GCF. Thus, experimental studies in rats and clinical studies in humans correlate alveolar bone remodeling with changes in GCF phosphatase activities [40, 45–47].

Various investigations consider the duration of an orthodontic cycle of 21 days, to identify and understand the enzymatic changes occurring during early stages of orthodontic force application in coincidence with initial and lag phases of tooth movement. It was observed that ALP activity peaked on the 14th day in most patients, followed by a sharp fall by the 21st day. The fall in activity is related to removal of the hyalinized zone. In hard bony tissues ALP is implicated in mineralization process because of the intense staining reaction given by active osteoblasts and osteocytes. No enzyme activity is found in bone matrix, except in close association with matrix synthesizing cells. The osteogenic cells in the periodontal ligament respond to tensional forces with an increase in the maturation rate. In periodontal ligament fibroblast and collagen proliferation are shown to increase under tension stress. ALP activity is low in the compressed hyalinized zones of the periodontal ligament; conversely ACP activity is higher. A late phase of bone deposition (7–14 days) occurs in both tension and pressure sites of the alveolar wall.

Thus, the predominant bone remodeling activity at the early times in a bone remodeling cycle is resorptive with increased acid phosphatase activity, but in the later phase resorption and deposition become synchronous. High levels of ALP have been described after 7 days, when bone deposition begins, and a significant peak occurs on day 14. It is obvious that, as a forerunner to bone formation, the number of fibroblasts and osteoblasts raise in tension areas. This amount occurs as a result of the increase in cell number by mitotic cell division. In histologic studies it has been observed that in marginal tensional areas cell proliferation occur between 36 and 50 hrs and lasts for 10 days or 3 weeks. On the compression side bone resorption would occur and osteoclastic activity would be high with little or no osteoblastic activity.

In conclusion, the analysis of the association between ALP and bone metabolism, under healthy gingival conditions, is a suggestive indicator of histological and biochemical changes in bone turnover and therefore of the amount of tooth movement.

Finally, specific properties of GCF ALP activity render this enzyme an interesting diagnostic tool in orthodontics.

## 3. Conclusions and Clinical Relevance

When exposed to varying degrees of magnitude, frequency, and duration of mechanical loading, alveolar bone and adjacent periodontal tissues show extensive macroscopic and microscopic changes.

Mechanical loading also alters periodontal tissue vascularity and blood flow, resulting in the local synthesis and release of various molecules, such as cytokines, growth factors, colony-stimulating factors, enzymes, and neurotransmitters. On the basis of sequential reactions and released substances, several of these biologically active molecules have been proposed as biomarkers to better understand the biological process involved in orthodontic tooth movement, to improve treatment and reduce adverse side effects [48]. A biomarker is a substance measured and evaluated objectively as an indicator of physiologic processes, pathogenic processes, or responses to a therapeutic treatment [49]. The biological mechanisms controlling the shift from the stimulus, consisting of continuous force application, to the reaction, represented by the displacement of the tooth in the periodontal space, could be evaluated by monitoring the higher or lower rate of such biomarkers in periodontium.

Potential biological markers can be collected by means of different experimental and clinical methods, respectively, in animal and in living human models. GCF analysis, especially, has offered several advantages for its simple, quick, and noninvasive collection and for the wide variety of molecules detected in his volume (Table 1).

Knowledge of the ongoing process occurring in periodontal tissues during orthodontic and orthopedic therapies can lead to proper choice of mechanical loading with the aim of shortening the period of treatment and avoiding adverse consequences associated with orthodontic treatment, such as root resorption or bone loss.

## Figures and Tables

**Table 1:** GCF biomarkers considered in the text and their biological significance.

Biomarkers of inflammation	
Interleukins (IL-1*β*, IL-6, IL-8)	
Tumor Necrosis factors (TNF-*α*)	
Colony-stimulating factors (M-CSF, G-CSF, GM-CSF)	
Growth factors (VEGF)	
Arachidonic acid derivates and prostaglandins (PGE)	
Calcitonin gene related peptide (CGRP)	
Substance P	
* Neutrophil* alkaline phosphatase (ALP)	
Biomarkers of bone resorption	
Receptor activator of nuclear factor kappa-B (RANK)	
Receptor activator of nuclear factor kappa-B ligand (RANKL)	
Biomarkers of cell death	
Caspase-1	
*Β*-glucuronidase (*β*G)	
Aspartate aminotransferase (AST)	
Lactate dehydrogenase (LDH)	
Biomarkers of bone deposition and mineralization	
Osteoprotegerin (OPG)	
* Bone *alkaline phosphatase (ALP)	
